# Preparation of Polydopamine Functionalized HNIW Crystals and Application in Solid Propellants

**DOI:** 10.3390/polym16111566

**Published:** 2024-06-01

**Authors:** Fengdan Zhu, Chang Liu, Desheng Yang, Guoping Li

**Affiliations:** School of Materials Science and Engineering, Beijing Institute of Technology, Beijing 100081, China

**Keywords:** polydopamine, functionalized crystals, HNIW, propellants

## Abstract

The application of hexanitrohexaazaisowurtzitane (HNIW) as an oxidizer in solid propellants aligns with the pursuit of high-energy materials. However, the phase transformation behavior and high impact sensitivity of HNIW are its limitations. Due to the strong adhesion and mild synthesis conditions, polydopamine (PDA) has been employed to modify HNIW. However, the method suffers from a slow coating process and a non-ideal coating effect under short reaction time. Herein, oxygen-accelerated dopamine in situ polymerization coating method was developed. It was found that oxygen not only reduced the coating time but also contributed to forming a dense and uniform PDA layer. HNIW@PDA coated in oxygen for 6 h exhibited the most favorable performance, with a delay of 20.8 °C in the phase transition temperature and a reduction of 145.45% in the impact sensitivity. The -OH groups on the surface of PDA enhanced the interaction between HNIW and polymer binders, resulting in a 20.36% reduction in the dewetting percentage. The lower content of PDA in HNIW@PDA (1.17%) resulted in minimal variation in the heat of explosion for HNIW@PDA-based HTPB propellant (6287 kJ/kg) in comparison to HNIW-based HTPB propellant (6297 kJ/kg). Hence, HNIW@PDA-based propellants are expected to offer an alternative with promising safety and mechanical performance compared to existing HNIW-based propellants, thus facilitating the application of HNIW in high-energy propellants. This work presents a low-cost method for efficiently inhibiting the phase transformation of polycrystalline explosives and reducing the impact sensitivity. It also offers a potential approach to enhance the interfacial interaction between nitro-containing explosives and polymer binders.

## 1. Introduction

Hexanitrohexaazaisowurtzitane (HNIW), with high heat and rapid energy release, is one of the most powerful non-nuclear explosives used in current applications [1]. Owing to the absence of halogen, HNIW can serve as an oxidizer in solid propellants (SPs), in line with the development trends of high energy and low characteristic signal of SPs [2,3]. Nevertheless, a high impact sensitivity and phase transition behavior have limited the application of HNIW in SPs [4,5]. The impact energy of HNIW is 5.6 J, which is 32% lower than that of cyclotetramethylene tetranitramine (HMX) [6]. It is partly due to the highly strained bond angles of HNIW and also due to the fact that microdefects on the surface of HNIW crystals are susceptible to form “hot spots” upon external stimulation, resulting in poor safety [7,8]. In terms of thermal properties, HNIW exhibits four phases at ambient temperature: α, β, γ, and ε [9]. Among them, ε-HNIW exhibits the highest crystal density (theoretical density of up to 2.04–2.05 g/cm^3^), the best thermal stability, and the lowest sensitivity [10]. However, at 160 °C~170 °C, ε-HNIW transforms into γ-HNIW, via phase transformation, leading to a decreased thermal stability and increased impact sensitivity. In addition, HNIW undergoes volume expansion during the phase transition, resulting in microdefects, which indirectly increases the impact sensitivity of HNIW [11,12]. Therefore, the high impact sensitivity and phase transition behavior have made HNIW less safe for storage, processing, and use, which constrain its application in SPs.

The construction of a core-shell structure with HNIW as the core and an inert layer as the shell is an effective way to inhibit the phase transformation and reduce the impact sensitivity [13,14]. Materials such as waxes, thermoplastic polymers, and graphite/graphene are commonly associated with the ability to form smooth and flexible films that prevent the formation and propagation of “hot spots” [15,16]. However, they are deficient in terms of excessive layer thickness, leading to a significant reduction in energy density. Low sensitivity explosives such as TATB has been adopted to coat HNIW to improve the safety and maintain the high energy density [17]. Nevertheless, the inadequate adhesion of TATB to polymer binders results in the suboptimal mechanical properties of composites when applied in SPs. Consequently, it is necessary to prepare thin, inert, and strongly adherent surface coating layer to meet the safety and energy requirements of HNIW in practical applications.

Polydopamine (PDA) is a material derived from the oxidative self-polymerization of dopamine (DA) under mild and controlled conditions [18,19]. The molecular structure of DA is shown in Figure S1. Since the existence of catechol and amino groups, PDA can form covalent and non-covalent interactions with the substrates [20]. Thus, PDA can firmly adhere to the surface of almost all materials such as ceramics, metals, and organics [19,21].

However, the polymerization process of DA is slow, and the formation of the PDA layer in air usually takes 10–24 h [22]. According to a report, PDA was employed to modify HNIW with a 12 h coating process, resulting in a 93.81% decrease in impact sensitivity [23]. Zhang et al. [24] prepared HNIW@PDA in air for a duration of 24 h with the delayed phase transition temperature from 156.3 °C to 179.0 °C. The long production time requires the use of costly and time-consuming preparation techniques, which is a significant barrier to the industrial application of HNIW@PDA. Lin et al. [25] shortened the reaction time to 6 h, but the phase transition temperature of HNIW@PDA was less delayed, rising from 167.0 °C to 178.7 °C. Consequently, it is imperative to reduce the preparation time of HNIW@PDA and optimize the modification effect of PDA on HNIW to ensure a robust foundation for the utilization of HNIW@PDA in SPs. In addition, HNIW@PDA exhibits reduced impact sensitivity and delayed phase transition temperature in comparison to pure HNIW, in accordance with the advancement of low sensitivity of SPs. Nevertheless, it remains to be demonstrated whether inert and -OH-containing PDA will affect the mechanical and energy performances of SPs. Therefore, in order to promote the wide application of HNIW in propellants, it is of great significance to explore the application of HNIW@PDA in SPs.

In this work, to effectively inhibit the phase transformation and improve safety performance of HNIW, oxygen-accelerated dopamine in situ polymerization coating method was developed to construct HNIW@PDA composites. The effects of the reaction atmosphere and duration on HNIW@PDA were investigated. The potential applications of HNIW@PDA in propellants were then explored, a topic that is rarely addressed in the existing literature. Finally, mechanical and energy properties of propellants with different contents of HNIW@PDA were examined.

## 2. Experimental

### 2.1. Materials

Dopamine hydrochloride and tris (hydroxymethyl) aminomethane (Tris-HCl) were provided by Aladdin Biochemical Technology Co., Ltd. (Shanghai, China). Hydrochloric acid (HCl), aqueous ammonia (NH_3_·H_2_O), sodium hydroxide (NaOH), acetone, and ethanol were purchased from Beijing TongGuang Fine Chemicals Company (Beijing, China). ε-HNIW (89 μm) was manufactured by Qing Yang Chemical Industry Corporation (Liaoyang, China). All utilized materials were of analytical grade.

### 2.2. Preparations of PDA and HNIW@PDA

**Scheme 1:**
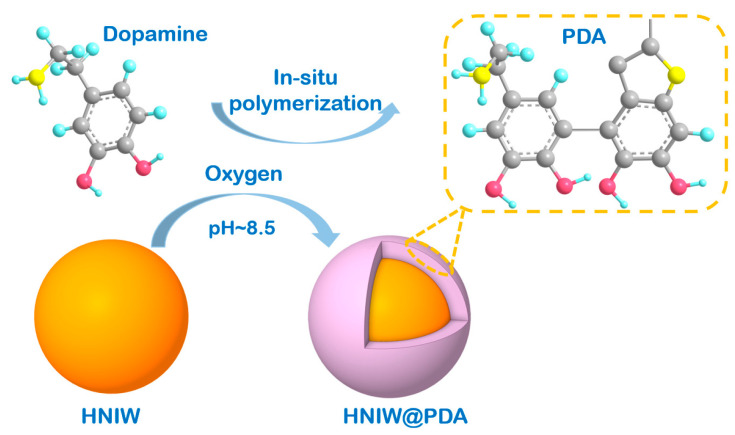
Description of the fabrication of HNIW@PDA.

The synthesis of pure PDA was explored in order to form a dense PDA layer on HNIW. PDA was prepared in three different systems: ethanol-NH_3_·H_2_O system, NaOH system, and Tris-HCl buffer system. Accordingly, PDA was designated as PDA-1, PDA-2, and PDA-3, respectively. In situ polymerization method was employed to prepare HNIW@PDA, with coating durations of 3, 6, and 9 h in air or oxygen. These samples were designated as HNIW@PDA-Air-x h and HNIW@PDA-O_2_-x h, in which x represents the coating time. The mechanism of DA in situ polymerization on HNIW is displayed in Scheme 1. The specific procedures of PDA and HNIW@PDA are listed in the Supporting Information. In order to clarify the existence type of PDA on HNIW, a dissolution verification experiment was carried out, utilizing the behavior that HNIW is soluble in acetone and PDA is insoluble in acetone. The details about the preparation are listed in the Supporting Information.

### 2.3. Preparation of Solid Propellants

The pouring process was adopted to produce SPs. Pure HNIW was partially or fully replaced by HNIW@PDA, and thus, SPs with different HNIW@PDA contents were prepared as shown in Table 1. SP-0 and SP-100% are designated as HNIW-based propellant and HNIW@PDA-based propellant.

Details concerning the characterizations of samples can be found in the Supporting Information.

## 3. Results and Discussions

### 3.1. Morphology and Structure of PDA

**Figure 1:**
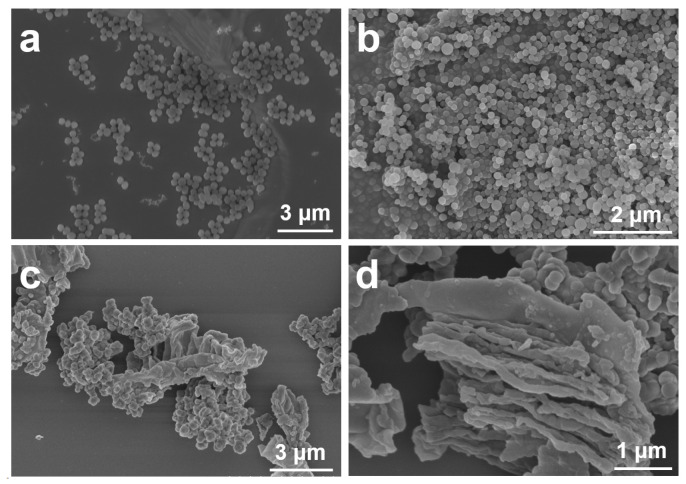
SEM images of (**a**) PDA-1, ethanol–NH_3_·H_2_O system; (**b**) PDA-2, NaOH system; and (**c**,**d**) PDA-3, Tris-HCl buffer system.

**Figure 2:**
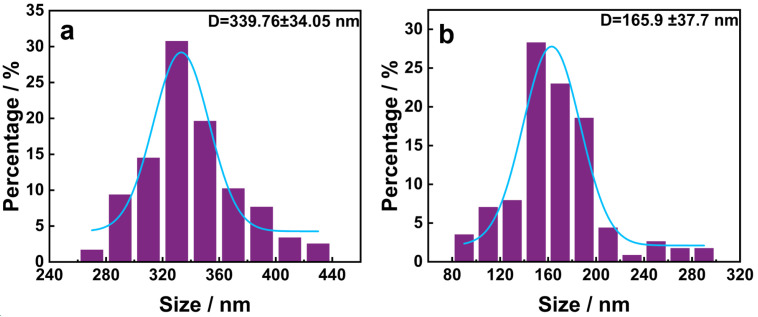
Size distribution of (**a**) PDA-1 and (**b**) PDA-2.

Scanning electron microscopy (SEM) was employed to observe the morphology of pure PDA prepared in three systems, as illustrated in Figure 1. Both PDA-1 and PDA-2 are submicron spherical particles, and the particle size distributions are shown in Figure 2. The synthesize time for PDA-1 is longer than that for PDA-2. The reason is that ethanol can quench the free radical activity, thus inhibiting the polymerization of DA [26]. PDA-3 is an aggregate with mutual adhesion, which is more likely to form a dense coating layer. Given the solubility of ethanol for HNIW [11], the Tris-HCl system is ultimately selected as the optimal choice for preparing HNIW@PDA crystals.

In order to elucidate the chemical structure of DA before and after polymerization, the infrared spectra of DA and PDA were examined by Fourier transform infrared spectroscopy (FT-IR) technique. The results are presented in Figure S2. Further discussion on the FT-IR spectra of PDA and DA can be found in the Supporting Information. The absence of the peak at 1519 cm^−1^ for PDA in comparison to DA indicates that the -NH_2_ group has undergone a conversion to the -NH group, which is indicative of a Michael addition reaction for DA [27].

### 3.2. Microscopic Morphology and Structure of HNIW@PDA

#### 3.2.1. Microscopic Morphology of HNIW@PDA

**Figure 3:**
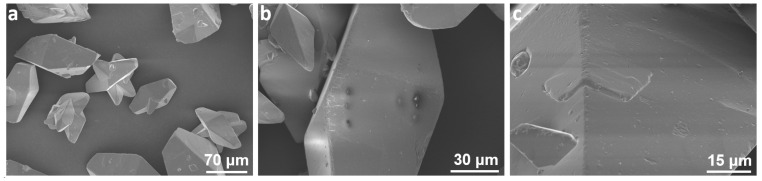
SEM images of raw HNIW at various magnifications of (**a**) 300; (**b**) 815; and (**c**) 1500.

**Figure 4:**
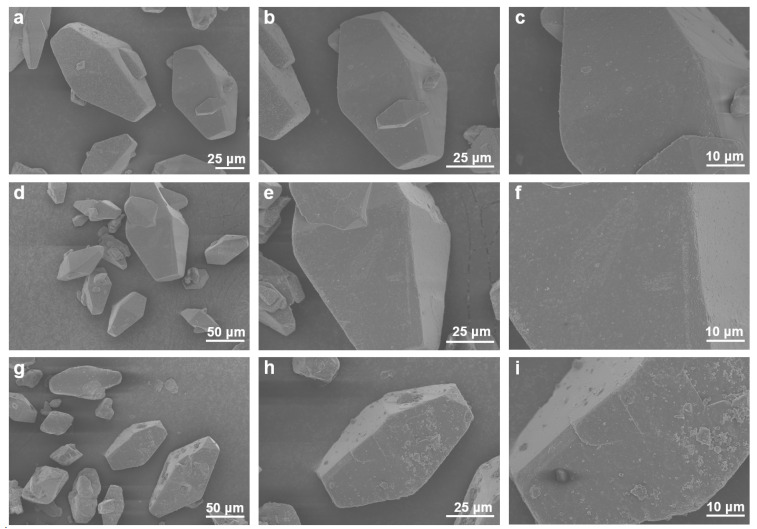
SEM images of (**a**–**c**) HNIW@PDA-Air-3 h; (**d**–**f**) HNIW@PDA-Air-6 h; and (**g**–**i**) HNIW@PDA-Air-9 h.

**Figure 5:**
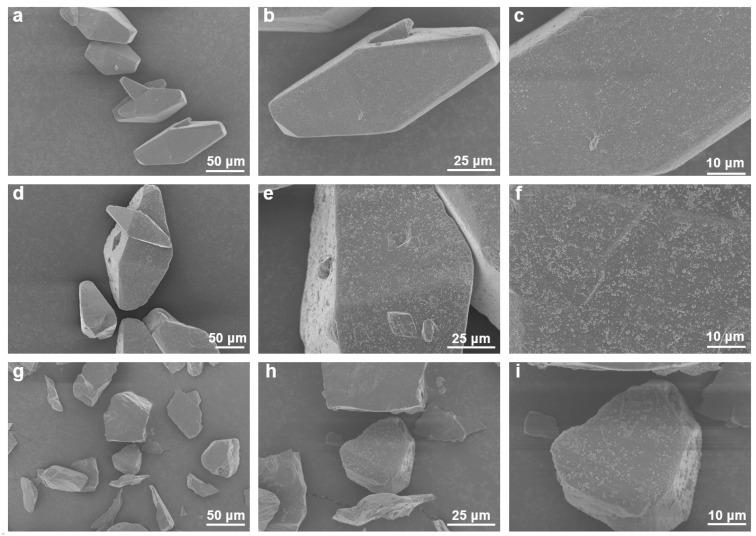
SEM images of (**a**–**c**) HNIW@PDA-O_2_-3 h; (**d**–**f**) HNIW@PDA-O_2_-6 h; and (**g**–**i**) HNIW@PDA-O_2_-9 h.

SEM was conducted to explore the effects of coating atmosphere and duration on the morphology of HNIW@PDA, as displayed in Figure 3, Figure 4 and Figure 5.

As illustrated in Figure 3, raw HNIW exhibits a spindle microform with a smooth surface. As depicted in Figure 3b, HNIW is highly sensitive to electron beam irradiation. It is easily damaged when exposed to the continuous assault of the electron beam for a slightly longer duration (within 4 s) at a magnification of 800~1000. As illustrated in Figure 4 and Figure 5, the surface of HNIW crystals coated with different durations in air and oxygen become rough. It is due to the agglomeration of PDA and the fact that PDA shells are deposited layer by layer on HNIW [28].

PDA particles on HNIW crystal surfaces are more densely packed with the extension of the coating time in both air and oxygen. However, when the coating time reaches 9 h, the shape of HNIW is no longer a complete spindle. It is due to the fragmentation of HNIW particles under prolonged high-speed mechanical agitation at 500 rpm. Therefore, in order to ensure the integrity and size distribution of HNIW particles, it is not advisable to coat for too long a period with a high rotational speed.

At a specific coating duration, HNIW coated in air exhibits a sparser PDA particle distribution than that coated in oxygen. Additionally, the uniformity of interparticle coating is superior in oxygen, as illustrated in Figure 4a,d. Despite the presence of HNIW in PDA particle deposits on the surface, the surrounding HNIW particles retain a relatively smooth appearance. However, as illustrated in Figure 5a,d, the surface of HNIW coated in oxygen not only exhibits dense PDA particles but also performs the tight coating of the edges, corners, and multiple surfaces, with no evidence of smooth HNIW. It is notable that HNIW coated in oxygen shows a pronounced decline in sensitivity to the electron beam, yet it remains stable at a magnification of 2000. Thus, oxygen can expedite in situ coating of PDA and enhance the uniformity and integrity of the layer. Two protons and electrons produced by each DA will react with oxygen to produce H₂O, and the reaction is reversible [22]. The forward progress of Equation (1) is promoted by oxygen blowing, ultimately leading to a superior coating effect of PDA on HNIW.

(1) $$\frac{1}{2}O_{2}+2H^{+}+2e^{-}⇋H_{2}O$$

**Figure 6:**
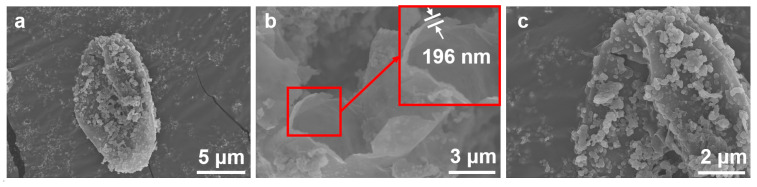
SEM images of residual PDA layer of HNIW@PDA-O_2_-6 h at various magnifications of (**a**) 5000; (**b**) 8000; and (**c**) 12,000.

To further confirm the coating integrity of PDA on HNIW, HNIW@PDA-O_2_-6 h was selected for acetone dissolution. The morphology of the residual PDA shells is presented in Figure 6. As shown in Figure 6a, following the dissolution and removal of nuclear HNIW, the remaining PDA shell maintains a spindle shape, with PDA particles persisting on the surface. Consequently, the existing PDA on HNIW are found to be complete dense layers rather than mere deposits. Figure 6b shows that the thickness of the PDA layer is approximately 200 nm.

#### 3.2.2. Structure of HNIW@PDA

**Figure 7:**
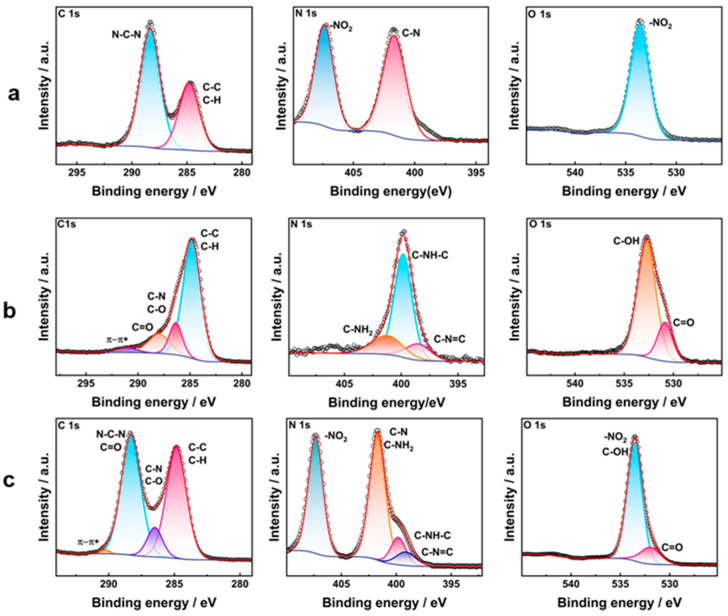
XPS spectra in arbitrary units (“a.u. units”) of (**a**) HNIW; (**b**) PDA; and (**c**) HNIW@PDA.

To verify the presence of PDA on HNIW, the surface chemical information of HNIW crystals before and after coating was analyzed by X-ray photoelectron spectroscopy (XPS). The Avantage software (version 5.938, Thermo Fisher Scientific, Waltham, MA, USA), as applied to conduct split-peak fitting after charge correction, as shown in Figure 7. The results of the peak of binding energy versus the chemical bonds are presented in Table S1.

As shown in Figure 7 and Table S1, C-C, C-H, C-N, C-O, C=O, C-NH_2_, C-NH-C, C-N=C, and C-OH are detected on the surface of PDA. The predominant chemical bonds on HNIW are N-C-N, C-C, C-H, C-N, and -NO_2_. HNIW@PDA shows different surface chemical information. For the C 1s spectrum, peaks of C=O, C-N, and C-O are newly observed compared to those for HNIW. For the N 1s spectrum, the adding peaks correspond to C-NH-C, C-NH_2_, and C-N=C. In the O 1s spectrum, only -NO₂ groups are present in HNIW, while C=O and C-OH are detected for HNIW@PDA. Therefore, the characteristic chemical bonds of PDA appear on the surface of HNIW@PDA compared to HNIW, indicating the presence of PDA on the surface of HNIW after coating. Furthermore, the presence of 5,6-hydroxyindole and 5,6-indole quinone in the spectra of PDA can be confirmed, which is consistent with previous findings [29].

Moreover, the peak area integration method was adopted to quantitatively analyze the percentage of atoms on the surface of the samples, and the results are shown in Table 2. HNIW, as a kind of high-energy material, contains a large number of -NO_2_ and C-N-C, resulting in a high surface N/C atomic ratio (1.02), while that of pure PDA is only 0.13. It is found that the N/C atomic ratio decreases from 1.02 to 0.62 after coating, which further confirms the formation of the PDA layer on the surface of HNIW.

**Figure 8:**
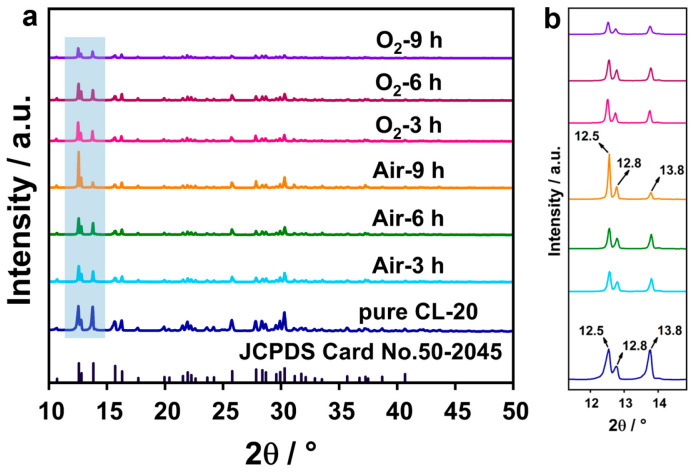
XRD patterns in arbitrary units (“a.u. units”) of HNIW and HNIW@PDA, (**a**) 10–50°; (**b**) 12–14°.

X-ray diffraction technology (XRD) was performed to determine whether the phase of HNIW is altered after being coated with PDA, and the diffraction patterns are illustrated in Figure 8. The positions and relative intensities of XRD pattern of raw HNIW are consistent with the standard pattern of ε-HNIW (JCPDS No. 50-2045). Peaks at 12.5°, 12.7°, 13.8°, 15.7°, 16.3°, 17.7°, 19.9°, 21.5°, 21.9°, 22.2°, 22.6°, 23.6°, 24.2°, 25.7°, 27.8°, 28.3°, 28.7°, 29.6°, 29.9°, 30.3°, 33.5°, and 35.7° correspond to the following crystal planes: (11-1), (011), (200), (120), (111), (021), (22-1), (211), (310), (130), (012), (13-1), (32-1), (022), (32-2), (40-2), (122), (42-1), (23-2), (20-3), (22-3), and (510), respectively [16]. The diffraction patterns of HNIW@PDA remain consistent with those of pure HNIW, indicating that the phase of ε-HNIW is not affected by the different durations of the coating process in air and oxygen. The region with the highest intensity of diffraction peaks (2θ = 12~14°) was selected for a thorough comparison, as shown in Figure 8b. Compared to ε-HNIW, γ-HNIW exhibits distinct peaks at both 2θ = 13.3° and 14.2° [30]. However, the two peaks are not observed in HNIW@PDA samples, suggesting that the coating process does not result in the transition of ε→γ.

**Figure 9:**
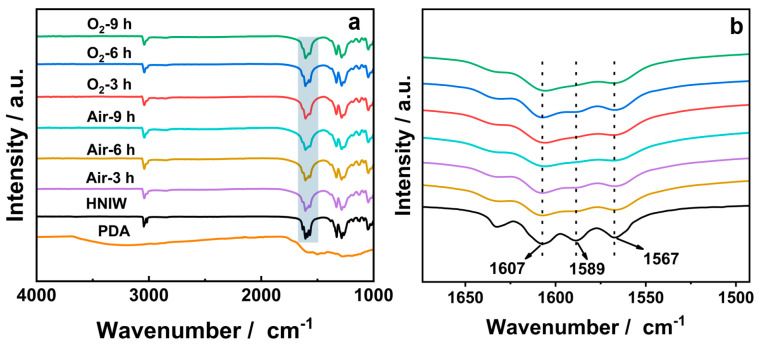
FT-IR spectra of PDA, HNIW, and HNIW@PDA, (**a**) 4000–1000 cm^−1^; (**b**) 1675–1500 cm^−1^.

FT-IR was conducted to ascertain the chemical structures of HNIW and HNIW@PDA, as shown in Figure 9. In the spectrum of HNIW, the triple peaks (1607 cm^−1^, 1589 cm^−1^, and 1567 cm^−1^) belong to the asymmetric stretching vibration of N-NO_2_ [31]. Since the peak positions of HNIW and HNIW@PDA are essentially identical, the molecular structure of HNIW is not altered by the coating technique. However, Figure 9b shows that the N-NO₂ bond intensity of HNIW@PDA is diminished in comparison to that of pure HNIW. The reason is that the PDA layer affects the absorption of IR light waves by HNIW. The peak at 1567 cm^−1^ of HNIW@PDA is shifted to a lower frequency, and the spectral band is broadened, which may be caused by the formation of hydrogen bonding between the -NO_2_ of HNIW and the -OH of PDA [32].

#### 3.2.3. Properties of HNIW@PDA

**Figure 10:**
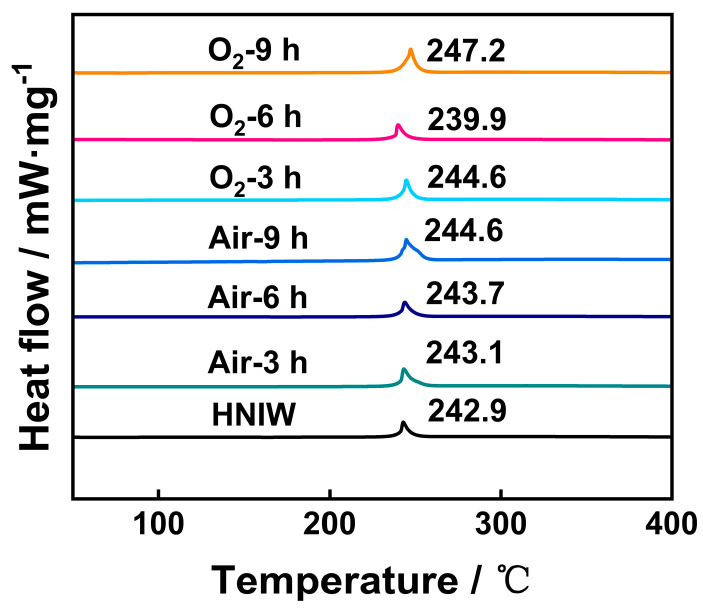
DSC curves of HNIW and HNIW@PDA.

Thermogravimetric differential scanning calorimetry (TG-DSC) tests were performed to obtain the thermal properties of HNIW@PDA, as displayed in Figure 10 and Table 3. The sharp exothermic peak at 242.9 °C represents the thermal decomposition peak temperature (*T_P_*) of HNIW [13]. *T_P_* of HNIW crystals subjected to coating in air and oxygen for 3, 6, and 9 h is similar to that of pure HNIW, with no significant decrease observed. Thus, HNIW and PDA exhibit good thermal compatibility [33].

The endothermic peak of pure HNIW at 163.9 °C (i.e., the phase transition peak temperature, *T_T_*) is due to the directional change in the -NO_2_ outside the cage structure [34]. Thus, the crystalline transforms from ε to γ phase induced by thermal stimulation [25]. *T_T_* of HNIW@PDA-Air-6 h, HNIW@PDA-O_2_-6 h, and HNIW@PDA-O_2_-9 h is 174.8, 184.7, and 171.5 °C, with 6.65%, 12.69%, and 4.64% increase, respectively, compared to that of pure HNIW. The complete and continuous PDA layer hinders the heat transfer during the crystalline transformation of HNIW. Furthermore, the strong interfacial interaction between PDA and HNIW can inhibit the rotation of -NO_2_ and the change in molecular conformation. It explains why PDA delays *T_T_* of HNIW [25,28]. Additionally, an appropriately extended coating time allows for the delay of *T_T_*. HNIW@PDA-O_2_-6 h exhibits the highest *T_T_*. However, *T_T_* of HNIW@PDA-O_2_-9 h is not delayed by the prolonged coating time. The findings are consistent with the previous SEM results, mainly due to the crystal fragmentation caused by the prolonged stirring duration. Similarly, no evident enhancement in *T_T_* of HNIW@PDA-Air-9 h is detected due to the impaired crystal integrity.

*T_T_* of HNIW@PDA-Air is typically lower than that of HNIW@PDA-O_2_, owing to the denser and more uniform PDA layer facilitated by oxygen. As a result, heat transfer is more effectively impeded by the PDA layer, and the change in molecular conformation is more strongly constrained for PDA-O_2_. Thus, oxygen-accelerated DA in situ polymerization coating technology represents a viable method for inhibiting the phase transformation of HNIW and other polycrystalline explosives. Optimal coating duration in oxygen is 6 h, which effectively delays *T_T_* of HNIW by 12.69%. A previous report [25] shows that *T_T_* of HNIW is improved 7.01% after a 6 h coating of PDA. Consequently, our work has demonstrated the capacity to enhance the modification effect of PDA on HNIW and to suppress the phase transformation of HNIW in a brief reaction time. This outcome can be attributed to the facilitation of oxygen on the reaction of electrons and protons during the polymerization process.

The impact sensitivity (IS) of HNIW reflects susceptibility to explosion upon impact [35]. A higher value of impact energy indicates a lower sensitivity to impact, which provides enhanced safety. In order to assess the effectiveness of the PDA layer in reducing IS of HNIW, HNIW and HNIW@PDA of varying preparation conditions were subjected to IS tests, with the results presented in Table 4.

The impact energy of pure HNIW is only 5.5 J. This is due to the fact that HNIW contains N-N bonds (with a dissociation energy of only 45.0 kcal/mol) [36], which are prone to decomposition due to heat accumulation when subjected to impacts. In terms of physical properties, the uneven area on the surface of the HNIW crystals can induce microbubbles, which can lead to heat accumulation [37]. In addition, HNIW undergoes phase transformation, resulting in new microgaps and other defects, which also evolve into “hot spots” [38]. The impact energy of HNIW@PDA is higher than that of HNIW due to the ability of the PDA layer to fill the uneven areas on the surface. In addition, the PDA layer suppresses the formation of new “hot spots” by inhibiting the phase transition. Furthermore, the impact of external forces can be cushioned via the dense and continuous inert PDA shells [39]. Hence, HNIW desensitized by PDA primarily relies on the filling of defects, the cushioning of impacts, and the inhibition of phase transition.

The appropriate extension of the coating time is beneficial for improving the desensitizing effect of PDA on HNIW. A short coating time is sufficient for PDA to form a complete layer on the surface of HNIW, which is not efficient enough to fill the defective areas. When the coating time is 9 h, the IS of HNIW@PDA is not further reduced. It is mainly due to the increased defects by the prolonged agitation. At a given coating time, HNIW@PDA-O_2_ shows a higher impact energy than HNIW@PDA-Air, and thus, oxygen is likely to reduce the IS of HNIW. When oxygen is blown, the PDA layer is denser than that in air [22], which can fully fill the microdefects on the crystal surface and reduce the exposed area of HNIW, thus efficiently decreasing the IS of HNIW. The impact sensitivity of HNIW@PDA-O_2_-6 h is 145.45% lower than that of HNIW, exhibiting the lowest impact sensitivity among all of the samples. The IS of HNIW@PDA prepared by Chen using a reaction time of 12 h was 93.81% lower than that of HNIW [23]. Hence, the technique of oxygen-accelerated DA in situ polymerization coating allows for a discernible enhancement of the safety of HNIW with a relatively brief reaction time. It is beneficial for improving the safety of nitro-containing explosives and developing safe high-energy SPs with low energy consumption. In addition, the technique is expected to be applied to effectively coat other materials such as ceramics, metals, and metal oxides.

The crystalline form of HNIW@PDA-O_2_-6 h is still the *ε* phase and exhibits the lowest IS and the highest *T_T_*, which is of high relevance for its applications. Hence, HNIW@PDA-O_2_-6 h (abbreviated as HNIW@PDA) was subsequently applied in SPs.

In order to determine the content of the PDA layer in HNIW@PDA, the mass ratio of C for PDA and HNIW before and after coating were obtained by organic elemental analysis (OEA) test and denoted as *C_P_*, *C_C_*, and *C_Mix_*, respectively, and the results are shown in Table 5.

Therefore, the mass fraction (*w*) of the PDA layer can be expressed by Equation (2), which was calculated to be 1.17%.

(2) $$w=\frac{C_{Mix}-C_{C}}{C_{P}-C_{C}}$$

### 3.3. Properties of Solid Propellants

#### 3.3.1. Mechanical Performance

**Figure 11:**
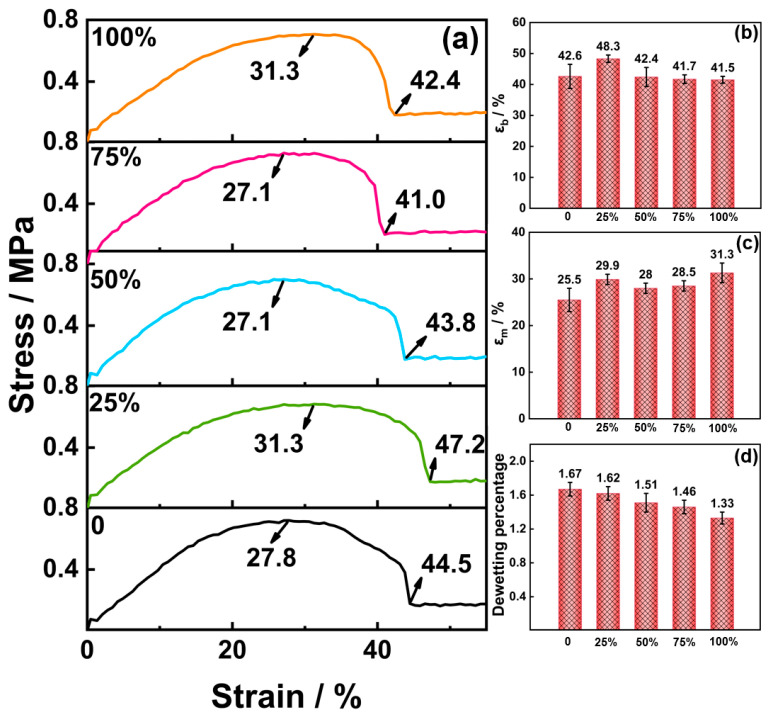
Mechanical performances of SPs: (**a**) stress–strain curve; (**b**) *ε_b_*; (**c**) *ε_m_*; and (**d**) average dewetting percentage.

For the HTPB/TDI adhesive system, the curing reaction occurs between -NCO and -OH groups [40]. Thus, -OH of PDA may affect the mechanical properties by influencing the curing reaction of SPs. Therefore, the mechanical properties of HNIW@PDA-based SPs were essential to be explored. Each SP sample was tested with 5~7 specimen rods, and one of the rods was selected to plot the stress–strain curve as shown in Figure 11a. According to the mechanical properties of 5~7 specimen rods, the average elongation at break (*ε_b_*), average maximum stress (*σ_m_*), and average maximum elongation (*ε_m_*) of the samples were obtained, as displayed in Figure 11b–d and Table S2.

As shown in Figure 11a, the stress–strain curves of SP-0, SP-25%, SP-50%, SP-75%, and SP-100% exhibit linear elasticity stage, dewetting stage, stress plateau, and fracture damage stage. Table S2 shows a similar *σ_m_* for all SPs (around 0.7 MPa), indicating that the tensile strengths of SPs are not negatively affected by the PDA layer on HNIW.

Dewetting is a type of interfacial damage that occurs when an external force disrupts the physical interaction between the solid particles and the polymer binders, resulting in separation between them [41]. Dewetting percentage is the ratio of *ε_b_* to *ε_m_*, which reflects the bonding state of the polymer binders to the solid particles [42,43]. Figure 11d shows that the dewetting percentage of SPs declines as the concentration of HNIW@PDA increases. The dewetting percentage of SP-0 is as high as 1.67, while that of SP-25%, SP-50%, SP-75%, and SP-100% decrease by 2.99%, 9.58%, 12.57%, and 20.36%, respectively. The catechol groups in PDA allow the formation of hydrogen bonds between HNIW@PDA and the carbamate bond of the polymer binders [44]. Hence, the strong adhesion and the improvement in the bonding strength contribute to the reduced dewetting percentage.

**Figure 12:**
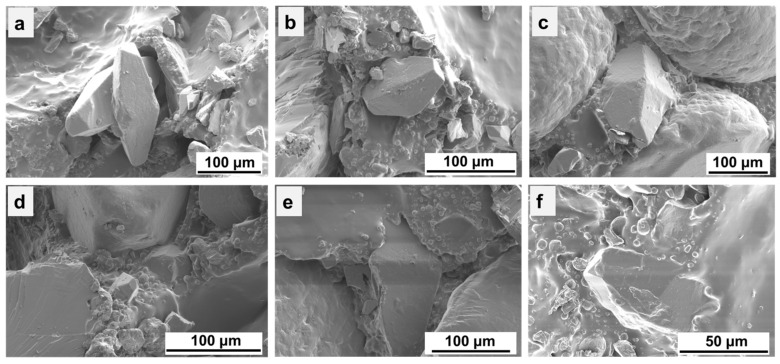
SEM images of tensile sections of (**a**) SP-0; (**b**) SP-25%; (**c**) SP-50%; (**d**) SP-75%; and (**e**,**f**) SP-100%.

For further insights into the dewetting state of SPs with varying HNIW@PDA contents, SEM was employed to observe the microscopic morphology of the tensile cross-sections, as presented in Figure 12. Spindle HNIW and HNIW@PDA crystals are observed in Figure 12. As shown in Figure 12a, no discernible coating on the surface of HNIW is observed in SP-0. HNIW crystals are uncoated and exhibit clear gaps with the polymer binders. HNIW crystals are not sufficiently in contact with the binders to form a robust interfacial bonding, leading to the heavy interfacial debonding. As the content of HNIW@PDA increases, a higher number of HNIW@PDA crystals can be found close to the binders, which is consistent with the results showing the gradual decrease in the dewetting percentage in Figure 11d.

Figure 12e,f demonstrates that HNIW@PDA is thoroughly coated with the polymer binders. The two-phase interface becomes less distinct but more blurred in SP-100%, and no significant debonding is presented owing to the strong adhesion of PDA [45]. Thus, microscopic observations and the reduced dewetting percentage indicate that the strong adhesion of PDA contributes to an enhanced interaction between HNIW@PDA and the polymer binders. Thus, in situ polymerization of DA offers a promising approach for enhancing the interfacial interaction between nitro-containing explosives and polymer binders.

#### 3.3.2. Energy Performance

The heat of explosion (*Q_V_*) is a crucial parameter for evaluating the energy performance of propellants. To assess the effect of PDA on the energy performance of propellants, oxygen bomb calorimetry was employed to assess the *Q_V_* of propellants in a confined nitrogen atmosphere. The results are presented in Table 6.

As illustrated in Table 6, the *Q_V_* of propellants with varying HNIW@PDA contents is basically consistent. The *Q_V_* of SP-100% exhibits a mere 0.16% reduction compared to SP-0. Therefore, *Q_V_* of the propellant is not affected by the substitution of HNIW@PDA for HNIW. It is due to the low content of inert PDA in HNIW@PDA, which is only 1.17%, that the mass fraction of PDA in the propellant is very low, and thus, *Q_V_* is basically unchanged.

## 4. Conclusions

In order to reduce the preparation time and improve the coating effect of PDA on HNIW, the oxygen-accelerated dopamine in situ polymerization coating method was developed to produce HNIW@PDA. Following a six-hour coating period, the phase transition temperature of HNIW@PDA prepared in oxygen was approximately 10 °C higher than that observed in air, while the impact energy was 3.5 J higher, demonstrating the most optimal performance. Moreover, the mechanical and energy properties of HTPB propellants containing HNIW@PDA were also investigated. Owing to the strong adhesion of PDA, an increase in the content of HNIW@PDA resulted in a reduction in the dewetting percentage of HTPB propellant. A 20.36% reduction in the dewetting percentage of HNIW@PDA compared to HNIW-based propellant was observed. It was evidenced by the microscopic morphology, which showed that the contact between HNIW@PDA and the polymer binders was close. Additionally, the low PDA content (1.17%) in HNIW@PDA did not result in a reduction in the heat of explosion of the HTPB propellant. This work offers a promising approach for effectively improving the safety of polycrystalline explosives and enhancing the interfacial interaction between nitro-containing explosives and polymer binders. Furthermore, the technique is anticipated to facilitate the modification of PDA on other materials such as metals, metal oxides, and polymers.

## Figures and Tables

**Table 1:** Formulation of SPs with different HNIW@PDA contents.

Sample	Contents of Component (wt%)							
	HTPB ^a^	TDI ^b^	DOS ^c^	HNIW	HNIW@PDA	AP ^d^	Al	T313 ^e^
SP-0	10.70	0.60	3.70	15.00	0	52.00	18.00	0.18
SP-25%				11.25	3.75			
SP-50%				7.50	7.50			
SP-75%				3.75	11.25			
SP-100%				0	15			

^a^, hydroxyl terminated polybutadiene, HTPB; ^b^, toluene di-isocyanate, TDI; ^c^, diisooctyl sebacate, DOS; ^d^, ammonium perchlorate, AP; and ^e^, boron trifluoride triethanolamine complex, T313.

**Table 2:** Surface percentage of atoms of HNIW, PDA, and HNIW@PDA.

Sample	C 1s (%)	N 1s (%)	O 1s (%)	N/C
HNIW	32.98	33.65	33.37	1.02
PDA	70.55	8.92	20.54	0.13
HNIW@PDA	43.24	26.98	29.78	0.62

**Table 3:** Thermal properties of HNIW and HNIW@PDA.

Sample	*T_T_* (°C)	*T_P_* (°C)
HNIW	163.9	242.9
HNIW@PDA-Air-3 h	162.4	243.1
HNIW@PDA-Air-6 h	174.8	243.7
HNIW@PDA-Air-9 h	161.0	244.6
HNIW@PDA-O_2_-3 h	168.1	244.6
HNIW@PDA-O_2_-6 h	184.7	239.9
HNIW@PDA-O_2_-9 h	171.5	247.2

**Table 4:** Impact energy of HNIW and HNIW@PDA.

Sample	Impact Energy (J)	Reduction Rate of IS (%)
HNIW	5.5	-
HNIW@PDA-Air-3 h	8.0	45.45
HNIW@PDA-Air-6 h	10.0	81.82
HNIW@PDA-Air-9 h	8.5	54.55
HNIW@PDA-O_2_-3 h	9.0	63.64
HNIW@PDA-O_2_-6 h	13.5	145.45
HNIW@PDA-O_2_-9 h	12.5	127.27

**Table 5:** Mass ratio of C for PDA and HNIW before and after coating.

Sample	Mass Ratio of C (%)
HNIW	16.76
PDA	53.52
HNIW@PDA	17.19

**Table 6:** *Q_V_* of SPs.

Sample	*Q_V_* (kJ/kg)
SP-0	6297
SP-25%	6289
SP-50%	6281
SP-75%	6292
SP-100%	6287

## Data Availability

Data are contained within the article and Supplementary Materials.

## Supplementary Materials

The following supporting information can be downloaded at: https://www.mdpi.com/article/10.3390/polym16111566/s1, Figure S1: Molecule structure of HNIW and dopamine; Figure S2: FT-IR spectra of DA and PDA; Table S1: XPS peak position of functional groups of HNIW, PA and HNIW@PDA; Tabel S2: *σ_m_* of SP.
