# Supplementary material for: Preparation of Polydopamine Functionalized HNIW Crystals and Application in Solid Propellants
Source: Polymers (Basel). 2024 Jun 1;16(11):1566. doi: 10.3390/polym16111566 (PMC11174638; doi:10.3390/polym16111566)
Supplement: Supplementary file 1 [file polymers-16-01566-s001.zip › polymers-3023457-supplementary.pdf]

Supporting Information

Preparation of polydopamine functionalized HNIW crystals and application in solid propellants

Fengdan Zhu[1], Chang Liu[1], Desheng Yang[1], Guoping Li[1*],

1. School of Materials Science and Engineering, Beijing Institute of Technology, Beijing, 100081, China

[*]**Corresponding author:** Guoping Li, E-mail: girlping3114@bit.edu.cn.

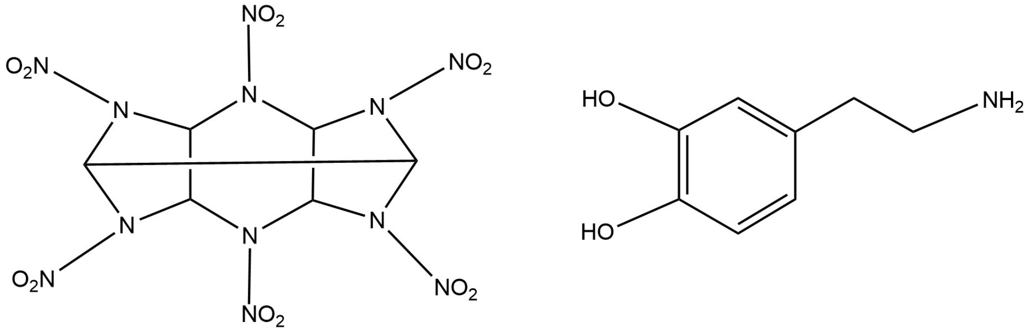

HNIW Dopamine

Figure S1. Molecule structure of HNIW and dopamine.

1. Preparations

1.1 Preparation of PDA

1.1.1 Ethanol-NH_3-H_2O System

32 mL of deionized water (DI) and 8 mL of ethanol were mixed well, to which 0.2 mL of ammonia was added dropwise. After stirring for 15 min, 0.1 g of dopamine hydrochloride (DA) was added and stirred at 25 ℃ for 6 h. The precipitate was separated by centrifugation (7000 rpm, 10 min) and washed with DI, and finally dried at 60 ℃ for 8 h to obtain PDA-1.

1.1.2 NaOH System

A solution of 0.36 g of DA in 180 mL of DI was prepared, to which 1.52 mL of a 1 mol/L NaOH solution was added. After stirring for 5 h at 50 ℃, the precipitate was separated by centrifugation (7000 rpm, 10 min) and washed three times with DI. Finally, the sample was dried at 60 ℃ for 8 h to obtain PDA-2.

1.1.3 Tris-HCl buffer system

A 0.01 M Tris-HCl solution was prepared and adjusted the pH to 8.5 via HCl, to which 0.6 g of DA was added. Then, the solution was stirred at 25 °C for 6 h with oxygen blowing. The black precipitate was obtained by centrifugation (7000 rpm, 10 min), washed three times using DI, and dried at 60 °C for 8 h to obtain PDA-3.

1.2 Preparations of HNIW@PDA and residual PDA shells

HNIW@PDA were prepared as follows: 2 g ε-HNIW crystals were added and dispersed in 60 mL Tris-HCl solution (10 mM, pH = 8.5). 0.12 g DA was introduced followed by stirring at 25 °C for 3, 6 and 9 h with air and oxygen blowing. Then, the composites were removed from the solution and thoroughly washed with DI 3-5 times, and finally dried at 60 °C for 8 h. The samples are noted as HNIW@PDA-Air-3 h, HNIW@PDA-Air-6 h, HNIW@PDA-Air-9 h, HNIW@PDA-O_2-3 h, HNIW@PDA-O_2-6 h, HNIW@PDA-O_2-9 h.

HNIW@PDA was added to excess acetone, sonicated for 10 minutes, and then stirred for 3 h. Centrifugation was employed to separate the supernatant from the precipitate (7000 rpm, 10 minutes), and after the supernatant was

poured off, acetone was continued to be added to the precipitate, sonicated, and centrifuged. This process was repeated five times to completely remove HNIW. The final precipitate was washed with ethanol and dried for 4-6 h at 60 ℃ to obtain the residual PDA shell from HNIW@PDA.

2. Characterizations

Scanning electron microscopy (SEM, Hitachi Regulus8230) was used to observe the morphology of samples. All samples were sprayed with platinum before testing and observed in high vacuum mode at an accelerating voltage of 3 kV. Fourier transform infrared spectroscopy (FT-IR) were recorded by Thermo Scientific Nicolet iS20 to evaluate the chemical structure of samples. X-ray diffraction (XRD, Rigaku SmartLab SE) was used to confirm the crystal structure of HNIW before and coated with PDA in the scanning angle from 10° to 50° at a rate of 2 °/min. X-ray photoelectron spectroscopy (XPS) was conducted by the monochromatic Al Kα line on Thermo Scientific K-Alpha to test the surface elemental information of samples. The impact sensitivity was tested in accordance with the European Commission 92/69/EEC, Method A14. BAM Fall Hammer (Idea Science Group, BFH-12) was used to test the impact sensitivity of HNIW and HNIW@PDA with a 10 kg hammer. Thermo Gravimetric Analyser with Differential Scanning Calorimeter (TGA-DSC) was performed on a Mettler Toledo instrument (TGA/DSC 3+). Approximately 1 mg of HNIW and HNIW@PDA samples were heated from 40 ℃ to 400 ℃ at a heating rate of 10 K/min under N_2 blowing. A total of 1~2 mg of the samples

was subjected to elemental composition analysis on an Elementar-UNICUBE type elemental analyzer (Elementar).

The mechanical properties of the SP were evaluated via an AGS-J electronic universal testing machine from Shimadzu, with a tensile rate of 100 mm/s. 5~7 strips were tested for each sample, and the mean maximum elongation(ε_m), elongation at break(ε_b), and mean maximum stress (σ_m) were calculated. A 0.9 g propellant was tested for heat of explosion by a Parr 6200 oxygen bomb calorimeter (Parr Instrument Company) in a closed nitrogen atmosphere with an initial temperature of 25 °C.

3. Results and discussions

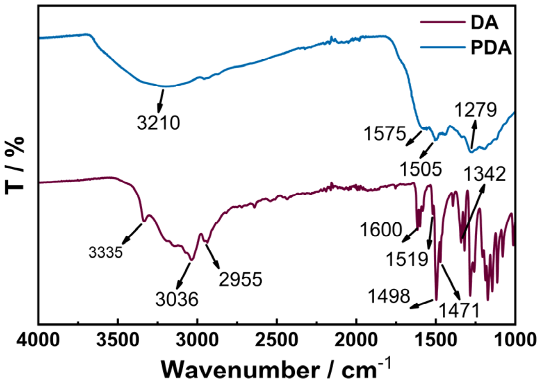

Figure S2. FT-IR spectra of DA and PDA.

For DA monomers, broader bands are observed in 3000-3400 cm^{-1}, which can be attributed to intermolecular hydrogen bonding. The peak at 3335 cm^{-1} belongs to the telescopic vibration of the primary amine group, while the peaks at 3036 and 2955 cm^{-1} are the asymmetric telescopic vibration of the phenolic hydroxyl group. The peak at 1342 cm^{-1} is caused by the bending vibration of -CH_2, while the peak at 1519 cm^{-1} corresponds to the shear vibration of the N-H of the primary amine group. The peaks at 1600, 1498, and 1471 cm^{-1} represents

the vibration of the aryl ring skeleton. For PDA, the peak at 1279 cm^{-1} corresponds to the asymmetric stretching and bending vibration of C-O. The shear and bending vibrations of the secondary amine moiety N-H lead to the peaks at 1505 cm^{-1} and 1575 cm^{-1}. The peak at 3210 cm^{-1} is characteristic of the phenolic hydroxyl O-H stretching vibration and N-H stretching vibration[1].

Table S1. XPS peak position of functional groups of HNIW, PDA and HNIW@PDA[2].

Sample	Atom	Peak position (eV)	Assignment
HNIW	C 1s	288.4	N-C-N
		284.8	C-C, C-H
	N 1s	407.4	$-NO_2$
		401.7	C-N
	O 1s	533.6	$-NO_2$
PDA	C 1s	288.0	C=O
		286.4	C-N, C-O
		284.8	C-C, C-H
	N 1s	401.3	$C-NH_2$
		399.9	C-NH-C
		398.6	C-N=C
	O 1s	532.8	C-OH
		530.9	C=O
HNIW@PDA	C 1s	288.3	C=O, N-C-N
		286.5	C-N, C-O
		284.8	C-C, C-H
	N 1s	407.4	$-NO_2$
		401.7	C-N, $C-NH_2$
		399.9	C-NH-C
		399.2	C-N=C
	O 1s	533.6	$-NO_2$, C-OH
		532.0	C=O

Tabel S2. σ_m of SP.

Sample	σ_m（MPa）
SP-0	0.728±0.012
SP-25%	0.687±0.015
SP-50%	0.697±0.020
SP-75%	0.739±0.016
SP-100%	0.710±0.027

4. References

1. Asha, A.B.; Chen, Y.; Narain, R. Bioinspired dopamine and zwitterionic polymers for non-fouling surface engineering. Chem. Soc. Rev. 2021, 50, 11668–11683.

2. Lin, C.; Yang, X.; He, G.; Wen, Y.; Qian, W.; Liu, R.; Liu, S.; Gong, F.; Zhang, J.; Zeng, C.; et al. Mussel-inspired interfacial reinforcement of thermoplastic polyurethane based energetic composites. Compos. Sci. Technol. 2023, 232, 109875.
